# Serum APN/CD13 as a novel diagnostic and prognostic biomarker of pancreatic cancer

**DOI:** 10.18632/oncotarget.12835

**Published:** 2016-10-24

**Authors:** Li Pang, Nan Zhang, Yan Xia, Dawei Wang, Guoqing Wang, Xiangwei Meng

**Affiliations:** ^1^ Department of Emergency, The First Hospital of Jilin University, Changchun 130021, China; ^2^ Department of Gastroenterology, The First Hospital of Jilin University, Changchun 130021, China; ^3^ Basic Medical College of Jilin University, Changchun 130021, China

**Keywords:** aminopeptidase N/CD13, pancreatic cancer, diagnosis, prognosis, biomarker

## Abstract

Aminopeptidase N, also known as CD13, has been reported to be overexpressed in several cancers and may contribute to tumor metastasis and angiogenesis. The aim of this study was to evaluate whether serum APN/CD13 could be a potential biomarker for the diagnosis and prognosis of pancreatic cancer (PC). Serum APN/CD13 and carbohydrate antigen 19-9 (CA19-9) levels were measured from 382 participants, which comprised of 204 participants with PC, 48 participants with benign pancreatic tumors (BPT), 43 participants with chronic pancreatitis (CP) and 87 healthy controls (HC). We used receiver operating characteristic (ROC) analysis to calculate diagnostic accuracy. The association of serum APN/CD13 levels with the clinicopathological characteristics of PC patients and their survival was investigated. Serum APN/CD13 levels were substantially higher in PC patients than in controls. ROC analysis revealed that APN/CD13 was significantly better than CA19-9 in differentiating patients with PC from controls. Similar results were noted for early-stage PC. Moreover, the combined use of APN/CD13 and CA19-9 data improved the diagnostic accuracy for PC *vs*. controls, compared with either test alone. High serum APN/CD13 levels were associated with tumor size, lymph nodes, and metastasis (TNM) stage. Multivariate and ROC curve analyses revealed that high serum APN/CD13 level is an independent factor for predicting mortality and overall survival (OS). Moreover, Kaplan–Meier analysis demonstrated an inverse correlation between increased serum APN/CD13 level and OS. Our study established that serum APN/CD13 may be a novel diagnostic and prognostic biomarker for PC.

## INTRODUCTION

Pancreatic cancer (PC) is one of the most highly aggressive types of malignant tumors, and the fourth leading cause of cancer-related deaths worldwide [1]. In spite of the development of surgical techniques and systemic treatment, due to local invasiveness and metastasis to distant organs at an early stage, the outcome of PC remains disappointing; and the average five-year survival rate is <5% [1, 2]. At present, the incidence and mortality rates of PC continue to increase, while early diagnosis of PC remains unsatisfactory. The standard serum marker in routine use for the management of PC is carbohydrate antigen 19-9 (CA 19-9). However, its inadequate sensitivity and specificity limit its use for the early diagnosis of PC [3]. The European Group on Tumor Markers (EGTM) reported that CA 19-9 could not be recommended for screening purposes, but only for monitoring response to therapy in patients who have elevated levels [4]. Several other biomarkers such as carcinoembryonic antigen (CEA) and carbohydrate antigen 242 (CA242) were identified as biomarkers for the diagnosis, prognosis and surveillance of PC. However, these markers are non-specific for PC and also lack sensitivity or specificity [5, 6]. Therefore, the identification of new and reliable noninvasive biomarkers is required for more accurate patient stratification and clinical care in PC patients.

Aminopeptidase N (APN/CD13), a Zn^2+^-dependent membrane-bound metalloproteinase, is a multifunctional cell surface ectopeptidase that preferentially degrades proteins and peptides with an N-terminal neutral amino acid [7]. APN/CD13 is expressed in many kinds of tissues and cells, and participates in extracellular matrix degradation, cell migration, angiogenesis and neoplastic invasion. APN/CD13 has been found to be overexpressed in most invasive human carcinomas, and a number of studies have suggested that APN/CD13 plays important roles in tumor progression, proliferation, tumor invasion and angiogenesis [8–10]. In addition, high expression levels of APN/CD13 in tumor tissues were found to correlate with increased malignant behavior and poor prognosis in prostate, colon, lung, thyroid, breast and pancreatic cancer [11–17]. High concentrations of serum/plasma APN/CD13 have been shown to be useful in the diagnosis of breast and thyroid cancer [18], and are associated with poor prognosis in non-small cell lung cancer (NSCLC) and colorectal cancer [19, 20]. However, the clinical significance of circulating APN/CD13 levels in PC has not yet been established.

In this study, we investigated circulating APN/CD13 levels in PC patients, assessed its diagnostic accuracy, and correlated its levels with clinicopathological features and survival rates.

## RESULTS

### Patient characteristics

A total of 204 PC (pancreatic adenocarcinoma) patients and 178 non-pancreatic cancer subjects including 48 benign pancreatic tumors (BPT) patients, 43 chronic pancreatitis (CP) patients and 87 healthy controls (HC) were recruited into this study. Their clinicopathological characteristics are summarized in Supplementary Table S1. Among the 48 BPT patients, 20, 16 and 12 patients had serous cystadenoma, tubular adenoma and intraepithelial neoplasia, respectively. These patients and controls were well-matched for age and gender overall.

Among the 204 PC patients, 130 were male and 74 were female; and median age of these patients was 67.4 years (range: 53-81 years). Based on the AJCC classification, 23 patients were diagnosed with stage I PC, 65 patients were diagnosed with stage II PC, 49 patients were diagnosed with stage III PC, and 67 patients were diagnosed with stage IV PC. We defined PC tumors with TNM stage T1 and T2 as early-stage PC. Accordingly, 88 (43.1%) of these PC patients were in the early-stage of the disease. Among these patients, 66 (32.4%) patients received radical surgery for pancreatic cancer, 55 (27.0%) patients received palliative surgery, and 83 (40.6%) patients did not receive surgery. Furthermore, among the 153 patients (75.0%) who received chemotherapy, 132 patients (64.7%) received chemotherapy alone and 21 (10.3%) patients received chemotherapy combined with local radiotherapy. Adjuvant chemotherapy was performed for surgical resection PC patients with 5-fluorouracil (5-FU)/leucovorin or gemcitabine. For PC patients with unresectable primary and/or distant metastatic tumors, we selected either gemcitabine based chemotherapy or 5-fluorouracil/leucovorin combined with irinotecan and oxaliplatin (FOLFIRINOX) chemotherapy. Gemcitabine based chemotherapy regimen includes gemcitabine monotherapy, gemcitabine plus erlotinib, gemcitabine plus capecitabine and gemcitabine plus cisplatin. The total radiotherapy dose was 50 - 54 Gy, each individual dose was 1.8 - 2.0 Gy.

### Serum levels of APN/CD13 and CA19-9 in PC patients and HCs and APN/CD13 expression in PC tissue

**Figure 1:**
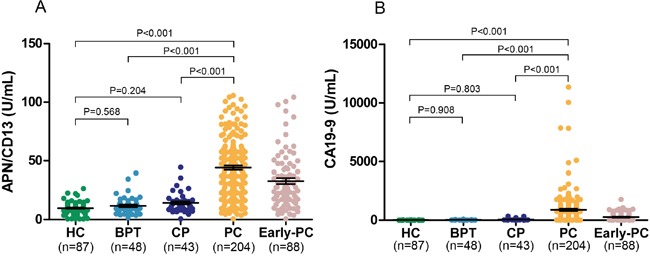
APN/CD13 and CA19-9 concentrations in serum of pancreatic cancer patients and in the control groups are shown Black horizontal lines indicate the mean, and error bars are standard errors. **A.** Serum APN/CD13, and **B.** serum CA19-9 levels. APN, Aminopeptidase N; CA19-9, carbohydrate antigen 19-9; HC, healthy control; CP, chronic pancreatitis; BPT, benign pancreatic tumors; PC, pancreatic cancer; SE, standard error. Statistical analysis was carried out using the Mann-Whitney U test.

Serum APN/CD13 concentrations were significantly higher in patients with PC than in controls (median 44.6 U/mL, interquartile range (IQR) 22.4–59.3 *vs*. median 9.9 U/mL, IQR 7.4–13.7; *P*<0.001; Figure 1, Supplementary Table S2), and these values did not significantly differ among the three control groups (Figure 1). Compared to the control groups, serum CA19-9 level was significantly higher in the PC group (median 633.4 U/mL, IQR 58.8–1082.0 *vs*. median 26.8 U/mL, IQR 11.4–40.3; *P*<0.001; Figure 1, Supplementary Table S2). There were no significant differences in serum concentrations of CA19-9 among the HC, CP and BPT groups (Figure 1).

In addition, we investigated the expression of APN/CD13 by immunohistochemical analysis in pancreatic tumor tissue specimens from 66 PC patients that underwent radical surgery. APN/CD13 protein was mainly found in association with cell membranes and cytoplasm (Supplementary Figure S1A). 51.5% (34 of 66) of the tumors stained positive for APN/CD13 expression (total immunoreactive score [IRS]: median 8, range 4-12) (Supplementary Figure S1A), while 48.5% (32 of 66) were negative for APN/CD13 protein expression (IRS: median 2, range 0-3) (Supplementary Figure S1B). There was no significant association between APN/CD13 expression and patient age, gender, tumor location, tumor size, tumor status, nodal status, metastatic status, or TNM stage (data not shown).

### Serum APN/CD13 is a sensitive marker to detect PC and early-stage PC

**Figure 2:**
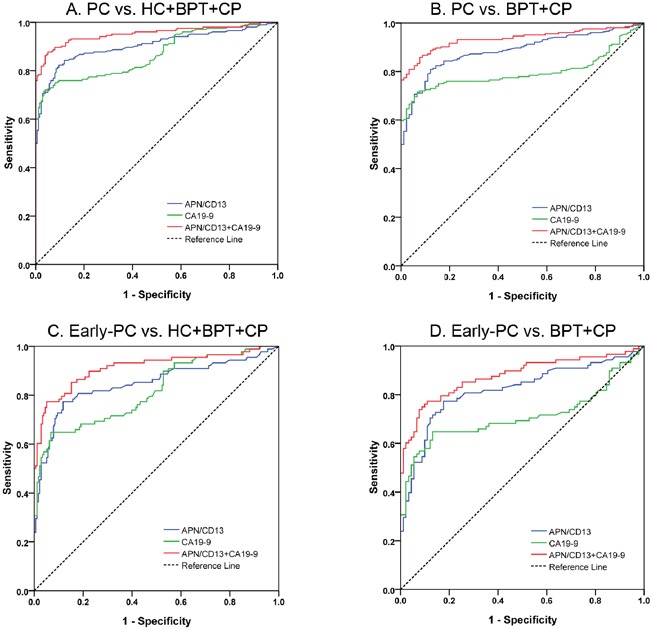
ROC analyses of serum APN/CD13 and CA19-9 in the diagnosis of PC or early-PC vs. non-malignant controls **A.** ROC curve for APN/CD13, CA19-9, or both for all patients with PC *vs*. all controls. **B.** ROC curve for APN/CD13, CA19-9, or both, for patients with PC *vs*. BPT and CP. **C.** ROC curve for APN/CD13, CA19-9, or both for patients with early-PC *vs*. all controls. **D.** ROC curve for APN/CD13, CA19-9, or both for patients with early-PC *vs*. BPT and CP. ROC, receiver operating characteristics; APN, Aminopeptidase N; CA19-9, carbohydrate antigen 19-9; HC, healthy control; CP, chronic pancreatitis; BPT, benign pancreatic tumors; PC, pancreatic cancer.

ROC curves revealed that the optimum diagnostic cutoff for APN/CD13 was 16.8 U/mL (AUC: 0.904, 95% confidence interval (CI): 0.872–0.936, sensitivity: 84.3%, specificity: 88.2%; Figure 2, Table 1). The optimum cutoff value for CA19-9 was 43.3 U/mL (AUC: 0.869, 95% CI: 0.833-0.905, sensitivity: 77.0%, specificity: 59.3%). Predictive values and likelihood ratios for APN/CD13 and CA19-9 in the diagnosis of PC are shown in Table 1. In the assessment of differential diagnostic accuracy, serum APN/CD13 had greater AUC, sensitivity, and specificity values compared with CA19-9 in the differentiation of patients with PC from controls (Figure 2, Table 1). All values increased when APN/CD13 and CA19-9 were combined (Figure 2, Table 1).

**Table 1:** Diagnostic parameters for serum APN/CD13, CA19-9, or the combination of APN/CA19-9, for the differentiation of pancreatic cancer (or early pancreatic cancer) from benign pancreatic tumors, chronic pancreatitis and healthy controls

	AUC (95% CI)	Sensitivity (%)	Specificity (%)	PPV (%)	NPV (%)	Positive LR	Negative LR
PC *vs.* BPT, CP and HC							
APN/CD13	0.904(0.872–0.936)	84.3	88.2	89.1	81.8	7.145	0.178
CA19-9	0.869(0.833–0.905)	77.0	59.3	80.5	78.6	1.892	0.388
APN/CD13+ CA19-9	0.952(0.930–0.975)	87.7	94.9	92.4	87.2	17.196	0.130
PC *vs*. BPT and CP							
APN/CD13	0.886(0.848–0.923)	84.3	82.4	89.1	66.7	4.790	0.191
CA19-9	0.796(0.746–0.847)	77.0	74.7	80.9	53.5	3.043	0.308
APN/CD13+ CA19-9	0.935(0.907–0.963)	88.7	87.9	91.9	75.8	7.331	0.129
Early-PC *vs*. BPT, CP and HC							
APN/CD13	0.847(0.789–0.904)	80.7	82.0	76.1	88.2	4.483	0.235
CA19-9	0.816(0.758–0.874)	69.3	70.2	60.8	82.8	2.326	0.437
APN/CD13+ CA19-9	0.913(0.870–0.955)	77.3	94.9	82.6	90.6	15.157	0.239
Early-PC *vs*. BPT and CP							
APN/CD13	0.819(0.753–0.884)	80.7	73.6	76.1	76.9	3.057	0.262
CA19-9	0.711(0.629–0.793)	69.3	52.7	61.5	65.1	1.465	0.583
APN/CD13+ CA19-9	0.877(0.824–0.931)	77.3	89.0	81.6	81.5	7.027	0.255

All parameters shown above were calculated for the optimum predicted probability for APN/CD13 (16.8 U/mL) and CA19-9 (43.3 U/mL). The optimum predicted probabilities of APN/CD13, CA19-9, and their combination were derived from their respective ROC curves by maximizing the sum of sensitivity and specificity and minimizing the overall error (square root of the sum [1-sensitivity]^2^ + [1-specificity]^2^), and by minimizing the distance of the cutoff value to the top-left corner of the ROC curve. APN, Aminopeptidase N; CA19-9, carbohydrate antigen 19-9; AUC, area under curve; PPV, positive predictive value; NPV, negative predictive value; LR, likelihood ratio; CI, confidence interval; HC, healthy control; CP, chronic pancreatitis; BPT, benign pancreatic tumors; PC, pancreatic cancer.

Among all PC patients recruited into this study, 88 (43.1%) of 204 patients were classified as early-stage disease (PC stages T1 + T2). APN/CD13 levels in serum were significantly higher in these patients than in all controls (*P*<0.001, Figure 1). Serum APN/CD13 improved the differential diagnosis of early-stage PC *vs*. all non-malignant controls, compared to CA19-9 (Figure 2, Table 1). The predictive values and likelihood ratios for APN/CD13 were also better than those for CA19-9 (Table 1).

ROC analysis revealed that testing of both APN/CD13 and CA19-9 increased the diagnostic accuracy for PC compared with either test alone (AUC: 0.952, 95% CI: 0.930–0.975, sensitivity: 87.7%, and specificity: 94.9%; Figure 2, Table 1). The diagnostic accuracy of the combination of APN/CD13 and CA19-9 remained improved even when only early-stage PC detection was assessed (Figure 2, Table 1).

### Serum APN/CD13 levels and clinicopathological characteristics in pancreatic cancer patients

Based on the diagnostic cutoff value of 16.8 U/mL for serum APN/CD13, PC patients were divided into two groups, with low (<16.8 U/mL) and high (≥16.8 U/mL) APN/CD13 levels. The relationship between serum APN/CD13 levels and various clinicopathological parameters of PC patients is summarized in Supplementary Table S3. High serum APN/CD13 levels in PC patients were associated with higher prevalence in all TNM stages (*P*=0.012). However, no statistically significant correlations among serum APN/CD13 levels and age, gender, tumor location, tumor size, tumor status, nodal status and metastatic status were observed.

**Figure 3:**
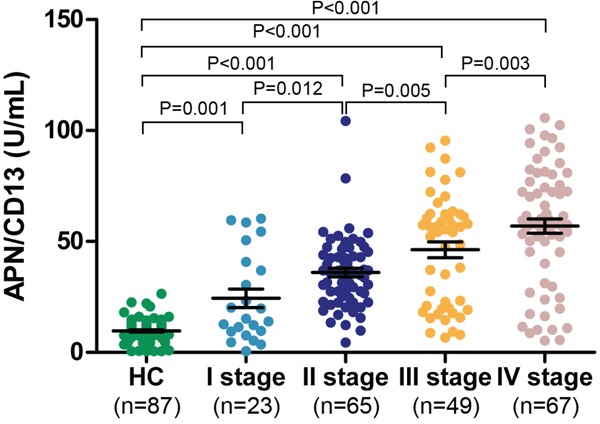
Serum APN/CD13 levels in patients with pancreatic cancer of different TNM stages vs. healthy controls Statistical analysis was carried out using the Mann-Whitney U test. HC: healthy controls.

**Figure 4:**
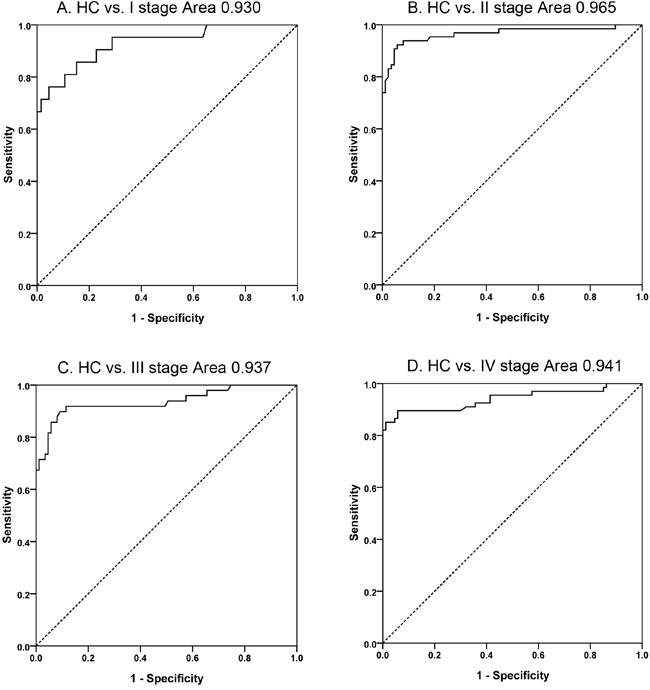
Diagnostic capabilities of APN/CD13 in differentiating pancreatic cancer patients with different stages vs. healthy controls **A.** ROC curve for patients with stage I pancreatic cancer *vs*. healthy controls. **B.** ROC curve for patients with stage II pancreatic cancer *vs*. healthy controls. **C.** ROC curve for patients with stage III pancreatic cancer *vs*. healthy controls. **D.** ROC curve for patients with stage IV pancreatic cancer *vs*. healthy controls. HC: healthy control; ROC: receiver operating characteristic.

Serum APN/CD13 levels in PC patients at any of the TNM stages were much higher than in HCs (Figure 3, Supplementary Table S4). Moreover, serum APN/CD13 levels significantly increased in high stage patients compared to low stage patients. Furthermore, AUCs of ROC curves for the differentiation between HCs and PC patients at different stages, based on APN/CD13 levels, were greater than 0.9; with sensitivities and specificities over 80% (Figure 4, Supplementary Table S5).

### Impact of serum APN/CD13 levels on one-year mortality

Eight PC patients were lost to follow-up at one year. Sixty-two of 196 patients died during the one-year period. Quantitative laboratory analyses revealed that serum APN/CD13 levels were higher in the non-survivors group compared with the survivors group (median 60.4 U/mL, IQR 54.4–73.6 *vs.* median 30.5 U/mL, IQR 18.2–47.5; *P*<0.001). Potential predictors of one-year mortality in PC patients are shown in Supplementary Table S6. Multivariate analysis determined serum APN/CD13 levels (OR: 3.35, 95% CI: 1.54–7.46, *P*=0.006), nodal status (OR: 2.52, 95% CI: 1.11–6.84, *P*=0.046), metastatic status (OR: 4.23, 95% CI: 1.96–8.35, *P*=0.026) and TNM stage (OR: 3.27, 95% CI: 1.63–8.10, *P*=0.013) as independent predictors of one-year mortality in PC patients. ROC analysis determined that serum APN/CD13 levels >49.9 U/mL predicted one-year mortality in PC patients with 87.9% sensitivity and 79.4% specificity (AUC: 0.877, 95% CI: 0.824–0.931; Supplementary Figure S2).

### Association between APN/CD13 and survival

**Figure 5:**
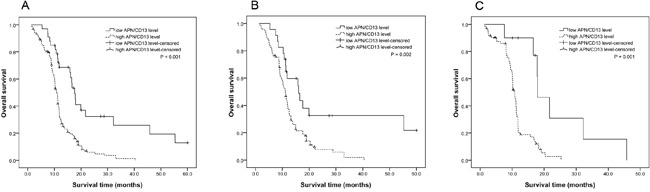
Survival curves for pancreatic cancer patients with different levels of serum APN/CD13 **A.** All PC patients. **B.** Pancreatic cancer patients with surgery. **C.** Non-operable pancreatic cancer patients. Serum APN/CD13 levels were bifurcated at the diagnostic cut-off value of 16.8 U/mL. Values ≥16.8 U/mL were considered high APN/CD13 levels, and values <16.8 U/mL were considered low APN/CD13 levels.

During the follow-up, median overall survival (OS) was 11.2 months (95% CI: 10.70–11.71) in all PC patients. Patients with low levels of serum APN/CD13 had better OS than patients who had high levels of serum APN/CD13 (*P*<0.001, Figure 5A). Median OS of patients with low levels of serum APN/CD13 was 17.6 months (95% CI: 15.99–19.21), while median OS of patients with high levels of serum APN/CD13 was 10.9 months (95% CI: 10.29–11.51). Moreover, these results revealed that high levels of serum APN/CD13 was significantly associated with shorter OS in PC patients with surgery (Figure 5B). Among non-operable PC patients treated with chemo regimens and/or radiotherapy, patients with high levels of serum APN/CD13 tended to have shorter OS (Figure 5C).

In addition, Cox regression analysis revealed factors that affect PC patient survival. Univariate Cox proportional hazard regression model analysis revealed that OS was significantly associated with age (*P*=0.043), nodal status (*P*=0.035), metastatic status (*P*=0.008), TNM stage (*P*=0.007), CA19-9 level (*P*=0.010) and serum APN/CD13 level (*P*=0.001) (Supplementary Table S7). Parameters significantly related to survival in the univariate analysis were entered into a multivariate analysis to identify independent factors for the prognosis of OS. Serum APN/CD13 level continued to maintain its significance as an independent prognostic factor for OS (HR: 3.12, 95% CI: 1.82–5.97, *P*=0.013) in PC patients. These results indicate that patients with high serum APN/CD13 have a higher risk of death. In addition, metastatic status, TNM stage and CA19-9 levels were identified as independent predictors of OS in PC patients (Supplementary Table S7).

## DISCUSSION

APN/CD13 is a type II transmembrane glycoprotein expressed in a variety of cells and tissues [7]. APN/CD13 participates in diverse pathophysiological procedures during cancer progression such as cell proliferation, invasion and metastasis [24]. Several studies [25, 26] have suggested that malignant cells with more highly expressed APN/CD13 degraded collagen more effectively; thus, impairing the basal membranes and extracellular matrix, and thereby promotes cancer cell invasion and metastasis. A study demonstrated that APN/CD13 could enhance invasive ability through interacting with tumor-associated surface antigen L6 [27]. Additionally, a number of studies [28, 29] have provided evidence that APN/CD13 plays a role in tumor progression by regulating cell–cell adhesion and angiogenesis.

The expression of APN/CD13 in tumor tissues has been found to be associated with poor prognosis in several malignancies. Hashida *et al*. reported that APN/CD13 was involved in cell motility or angiogenesis, and that the expression of APN/CD13 was associated with poor prognosis for node-positive patients with human colon cancer [12]. Studies on NSCLC demonstrated that the expression of APN/CD13 was associated with angiogenesis, TNM stage and lymph node metastasis status; and that patients with APN/CD13 positive tumors had a significantly lower five-year survival rate than patients with APN/CD13 negative tumors [13, 14]. APN/CD13 expression in breast cancer significantly correlated with neoangiogenesis, and OS tended to be shorter in patients with APN/CD13 positive tumors; which suggests that APN/CD13 expression can serve as an indicator of poor prognosis in the evaluation of breast cancer [16]. A recent study [30] found that strong APN/CD13 expression in hepatoblastoma correlated with vascular invasion, and event-free survival and OS were better in patients with low APN/CD13 than in patients with high APN/CD13 tumors. This indicates that APN/CD13 expression is associated with hepatoblastoma invasiveness, and suggests that APN/CD13 expression could be used as a prognostic marker for hepatoblastoma. APN/CD13 was also shown to be overexpressed in established human pancreatic carcinoma tissues, with a significant association between APN/CD13 expression and the increase in intratumoral microvessel density; and that APN/CD13 status is an independent predictive factor of survival in patients with pancreatic carcinoma [17].

All these findings underline the importance of APN/CD13 as a biomarker for tumor progression, metastasis and clinical outcome of some cancers. However, the molecular analyses of tumor tissues have some limitations due to their restricted availability, and the invasive characteristic of tumor biopsies. Therefore, the establishment of blood-based tests is important. Studies have shown that levels of circulating APN/CD13 can be measured quickly, and may reflect APN/CD13 expression in tumor tissues; which may provide a useful marker with diagnostic and prognostic significance in cancer patients [19]. A biomarker study conducted by Severini *et al*. revealed that an APN/CD13 enzymatic activity test based on serum samples could be useful in the diagnosis of breast and thyroid cancer [18]. Van Hensbergen *et al*. found that soluble APN/CD13 is elevated in plasma and effusions in cancer patients, compared with HCs. There is also a strong correlation between plasma APN/CD13 and tumor load, suggesting that plasma APN/CD13 at least partly originates from cells in, or related to, the tumor [31]. Murakami *et al*. demonstrated that high serum APN/CD13 levels were significantly associated with adverse prognostic factors such as advanced stage, poor performance status, and poor response to chemotherapy in NSCLC. Serum APN/CD13 levels were also associated with overall survival, and had an independent influence on the survival of patients with NSCLC [19]. A recent study demonstrated the potential of tissue and plasma APN/CD13 as an independent prognostic factor of five-year survival in colorectal cancer patients [20]. To date, the clinical significance of levels of circulating APN/CD13 in human PC remains to be established.

Our pilot study is the first report to examine the diagnostic and prognostic value of serum APN/CD13 levels in PC patients. We found that serum APN/CD13 levels were significantly elevated in patients with PC, compared to control subjects. Our data revealed that serum APN/CD13 represents a marker of high sensitivity and specificity for detecting PC, and that its diagnostic value is better than that of CA19-9. Moreover, serum APN/CD13 can differentiate between tumor stages of PC patients, and particularly identify early-stage PC patients; which could help in the early detection of PC and improve survival rates. This indicates that serum ANP/CD13 could be used as a reliable alternative to CA19-9 in the diagnostic performance of PC. In addition, the combined testing of both APN/CD13 and CA19-9 concentrations in serum could further improve these results. Increased soluble forms of APN/CD13 were also demonstrated in breast, thyroid, colorectal, ovarian, renal and lung cancer patients [18–20, 31, 32]; which indicate that APN/CD13 might have potential as a cancer-specific serum biomarker for various cancers including PC. Thus, the integration of the measurement of serum APN/CD13 into the diagnostic work-up for PC, together with information on CA19-9, tumor imaging and other clinicopathological features, should be considered.

We demonstrated that the levels of APN/CD13 in PC patients were associated with TNM stage. Similar results have also been reported in NSCLC [19]. Our study further demonstrated that elevated serum APN/CD13 levels had a high predictive value for one-year mortality. Moreover, patients with high levels of APN/CD13 had significantly shorter OS. Cox proportional hazard regression model analyses revealed that the level of APN/CD13 was an independent factor for the prediction of OS. These results are consistent with findings of circulating APN/CD13 in NSCLC and colorectal cancer [19, 20], and are also in line with earlier reports of APN/CD13 in established human pancreatic carcinoma tissues [17]. We demonstrated for the first time that high serum levels of APN/CD13 at the time of diagnosis of PC patients indicate poor prognosis. Thus, elevated levels of serum APN/CD13 could be used for risk stratification of PC.

Several limitations in the present study need to be addressed in the future. Firstly, serum APN/CD13 levels were measured only once before the initiation of treatment in the present study. The serial measurement of serum APN/CD13 levels would certainly provide valuable additional information. The second limitation is the size of the study groups, and the diagnostic and prognostic value of this biomarker needs to be validated in larger, prospective and controlled clinical studies.

In conclusion, our results demonstrate that serum APN/CD13 could be potentially used to diagnose PC, especially early-stage disease; and high serum APN/CD13 levels were associated with more advanced clinicopathological features and poor outcome in PC patients. Thus, it has the potential to be a valuable diagnostic and prognostic biomarker in PC.

## MATERIALS AND METHODS

### Study population

PC patients who had not undergone any form of antitumor therapy prior to admission at the First Hospital of Jilin University between January 2010 and December 2014 were consecutively enrolled into this study. Consecutive patients presenting at the same hospital with BPT or CP, as well as HC, were recruited as controls into this study. The diagnosis and clinical stages of PC were based on the 7^th^ edition of the American Joint Committee on Cancer (AJCC) staging system [21]. For the purpose of this study, tumors with TNM stage T1 and T2 were classified as early-stage PC. The diagnosis of CP was based on clinical, laboratory and imaging evidence (ultrasonography, CT, or MRI) [22]. The presence of BPT was confirmed by symptoms, imaging (CT or MRI), laboratory and pancreatic tumor biopsy. HC were eligible donors with no history of pancreatic disease, and no malignant disease. Patients who had a history of other solid tumors were excluded from the study.

Patients were registered in a prospectively-collected PC database, and were followed up every three months up to the death of the patient or until December 2015. OS was defined as the interval between of the initial treatment date and death of the patient due to PC or last follow-up. The study protocol was approved by the Medical Ethics Committee of the First Hospital of Jilin University, and written informed consent was obtained from the patients and healthy volunteers before enrollment in the study. The study protocol conformed to the ethical guidelines of the Declaration of Helsinki.

### Blood samples and measurement of serum APN/CD13 and CA19-9

Venous blood was collected from each patient at the time of diagnosis and from controls. Blood samples were collected into anticoagulant-free tubes. After clotting, these samples were centrifuged at 3,000 rpm for 10 minutes. Serum was collected and stored at −70°C until assayed. The assay was based on the APN/CD13–specific monoclonal antibody MH8-11 and the commercial anti-APN/CD13 monoclonal antibody MCA1270 (Bio-Rad). MH8-11 was produced by immunizing BALB/c mice with HT1080 cells, as previously described [12]. This served as the detection antibody, while MCA1270 was used as the capture antibody. The serum concentration of APN/CD13 was assayed using a modified version of the electrochemiluminescence immunoassay (ECLIA) method previously described [19]. Briefly, serum samples were incubated for 10 minutes with biotin (Thermo Scientific)-labeled MCA1270 and ruthenium (Gibco, Invitrogen)-labeled MH8-11, and incubated for 10 minutes with streptavidin-biotin magnetic particle buffer (Roche; particle concentration, 0.72 mg/mL). After incubation, the reaction mixture was drawn into the measuring cell, and the samples were magnetically captured on the electrode. After increasing the voltage on the electrode, the electrochemiluminescence response was initiated and recorded on the ECLIA analyzer (Roche). The assay was calibrated with culture supernatant derived from the human fibrosarcoma cell line HT1080. This standard was determined to have an APN/CD13 concentration of 128 U/mL. It was serially diluted in matrix buffer to yield concentrations of 64, 32, 16, 8, 4, 2, 1 and 0.5 U/mL. APN/CD13 concentrations were calculated from a standard curve determined by the concurrent determination of the standard. CA19-9 concentrations were measured using the sandwich ECLIA method (Roche, Elecsys 2010), according to the manufacturer's instructions. Briefly, serum samples were incubated with two monoclonal antibodies that bind to different epitopes of CA19-9. These antibodies were labeled with biotin and ruthenium complex, respectively. Then, streptavidin-coated microparticles were added to the samples. The reaction mixture was aspirated into the measuring cell where the microparticles were magnetically captured onto the surface of the electrode. Unbound substances were removed. Finally, the application of increased voltage to the electrode induced chemiluminescent emission, which was measured by an ECLIA analyzer (Roche). All measurements were performed in duplicate.

### Immunohistochemistry staining

Tumor tissues originated from 66 PC patients who underwent radical surgery. Briefly, paraffin-embedded tissue specimens were sectioned to 3.5 μm thickness and were deparaffinized, rehydrated, and subjected to antigen retrieval in a pressure cooker in citrate buffer. Sections were treated with 3% hydrogen peroxide to block endogenous peroxidase activity, and then stained with the APN/CD13–specific monoclonal antibody MH8-11 at a dilution of 1:100. After washing in PBS, horseradish peroxidase (HRP)-labeled anti-rabbit IgG was added as secondary antibody-enzyme conjugate. Finally, diaminobenzidine (DAB) was added and the specimens were counterstained with hematoxylin. Non-immune rabbit serum at the same dilution was adopted as the negative control. Staining results were evaluated independently by two pathologists without prior knowledge of clinicopathologic data. The staining intensity of APN/CD13 was graded from 0 to 3 (no staining = 0, weak staining = 1, moderate staining = 2, strong staining = 3). The distribution of positively stained cells was scored on a scale of 0-4: 0, no staining; 1, <25%; 2, 25-50%; 3, >50-75%; 4, >75%. The total immunoreactive score (IRS) was calculated as the staining intensity×distribution (score 0-3, negative expression and 4-12, positive expression). The IRS was used for the classification of specimens as either APN/CD13-positive or -negative.

### Statistical analysis

Statistical analysis was carried out using the Mann-Whitney U-test and Chi-square test. Receiver operating characteristics (ROC) analysis was used to qualify marker performance, and ROC curves were constructed to assess sensitivity, specificity, and respective areas under the curves (AUCs) with 95% CI. In the ROC curves the true positive rate (sensitivity) is plotted against the false positive rate (1-specificity) for different cutoff points of a parameter. The optimum cutoff value for diagnosis was chosen using the Youden index [23]. Cumulative survival rates were calculated by the Kaplan-Meier method, and differences were analyzed using the log-rank test. Multivariate Cox's proportional hazard analysis was carried out to compare and identify independent prognostic factors for OS, and to calculate hazard ratios (HR) and 95% CIs. All significant parameters determined in the univariate analysis were entered into the multivariate model. SPSS software (version 16.0) and GraphPad Prism software (version 5.01) were used for statistical analysis. All hypotheses were formulated as two tailed, and a *P*-value <0.05 was considered statistically significant.

## SUPPLEMENTARY MATERIALS FIGURES AND TABLES


