# Supplementary material for: Serum APN/CD13 as a novel diagnostic and prognostic biomarker of pancreatic cancer
Source: Oncotarget. 2016 Oct 24;7(47):77854–64. doi: 10.18632/oncotarget.12835 (PMC5363626; doi:10.18632/oncotarget.12835)
Supplement: Supplementary file 1 [file oncotarget-07-77854-s001.pdf]

Serum APN/CD13 as a novel diagnostic and prognostic biomarker of pancreatic cancer

SUPPLEMENTARY FIGURES AND TABLES

A B

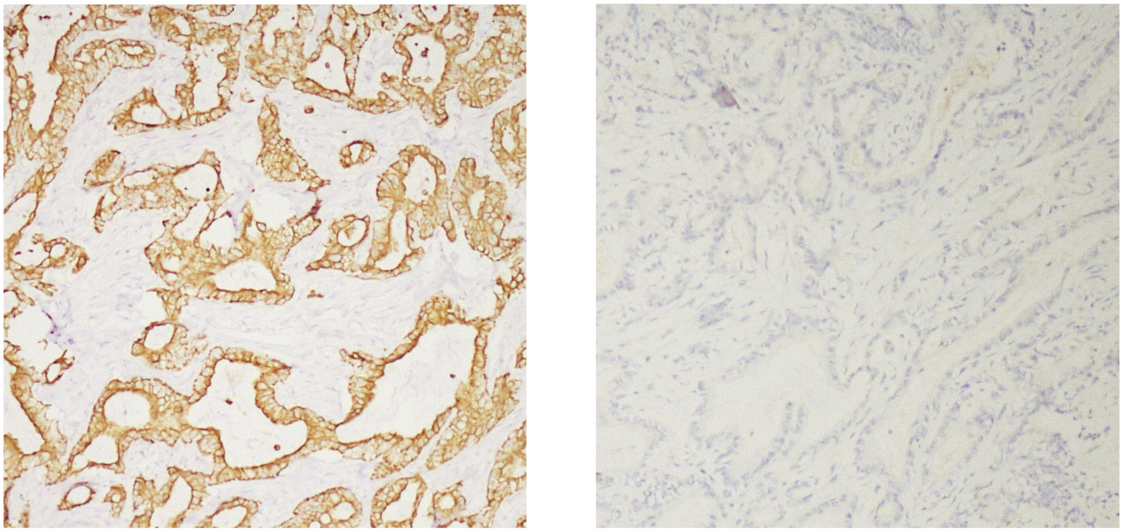

Supplementary Figure S1: Immunohistochemical analysis of the expression of APN/CD13 protein in pancreatic cancer specimens from patients who underwent radical surgery (magnification, ×100). Representative examples for APN/CD13-positive (**panel A**) and APN/CD13-negative (**panel B**) specimens are shown. Staining for APN/CD13 protein was mainly found in association with cell membranes and cytoplasm (panel A). 51.5% (34 of 66) of resectable tumors stained positive for APN/CD13 expression, while 48.5% (32 of 66) were negative.

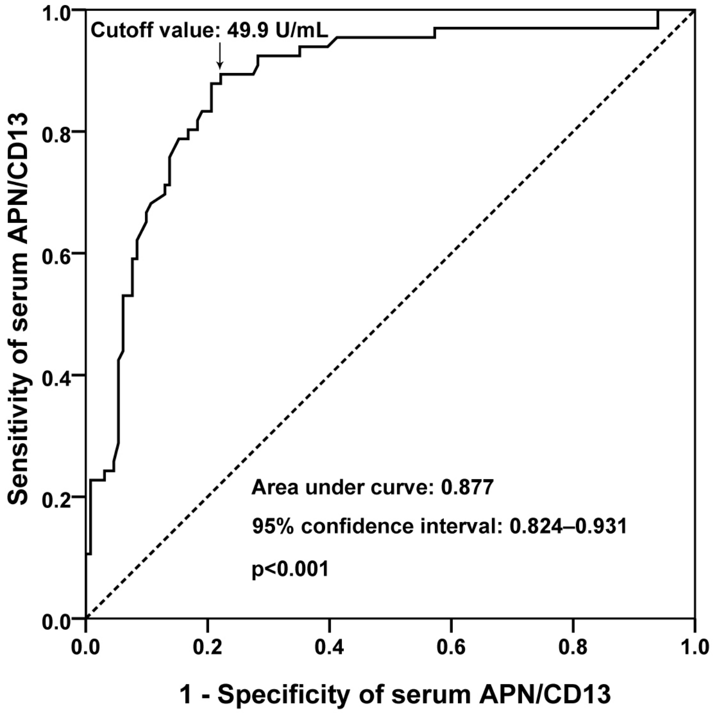

Supplementary Figure S2: ROC analysis for one-year mortality in pancreatic cancer patients predicted by the level of serum APN/CD13. The optimal cut-off point of 49.9 U/mL of APN/CD13 predicted the one-year mortality with 87.9% sensitivity and 79.4% specificity (AUC: 0.877, 95% CI: 0.824–0.931).

Supplementary Table S1: Clinicopathological characteristics of recruited participants (*n*=382)

Characteristic	Pancreatic Cancer *n* (%)	Benign pancreatic tumor *n* (%)	Chronic pancreatitis *n* (%)	Healthy controls *n* (%)
Age (y)				
≥60	126 (61.8)	28 (58.3)	26 (60.5)	56 (64.4)
<60	78 (38.2)	20 (41.7)	17 (39.5)	31 (35.6)
Gender				
Male	130 (63.7)	30 (62.5)	28 (65.1)	54 (62.1)
Female	74 (36.3)	18 (37.5)	15 (34.9)	33 (37.9)
Tumor location				
Head	149 (73.0)			
Body/tail	55 (27.0)			
Tumor size				
<5 cm	122 (59.8)			
≥5 cm	82 (40.2)			
Tumor status				
T1	50 (24.5)			
T2	62 (30.4)			
T3	40 (19.6)			
T4	52 (25.5)			
Nodal status				
N0	72 (35.3)			
N1	132 (64.7)			
Metastatic status				
M0	137 (67.2)			
M1	67 (32.8)			
TNM stage				
I	23 (11.3)			
II	65 (31.9)			
III	49 (24.0)			
IV	67 (32.8)			

Supplementary Table S2: Concentrations of serum APN/CD13 and CA19-9 in different study groups

Group	*n*	APN/CD13 (U/mL)		CA19-9 (U/mL)	
		Median (IQR)	Mean (SD)	Median (IQR)	Mean (SD)
Healthy controls	87	9.3 (6.6–12.4)	9.7 (4.8)	11.8 (8.2–17.7)	15.0 (10.4)
Chronic pancreatitis	43	12.4 (8.5–16.3)	14.2 (8.2)	45.6 (33.5–67.4)	64.5 (69.5)
Benign pancreatic tumor	48	9.7 (7.2–14.1)	11.7 (7.4)	37.9 (27.5–45.3)	37.2 (15.5)
Pancreatic cancer	204	44.6 (22.4–59.3)	44.1 (24.9)	633.4 (58.8–1082.0)	894.8 (1450.0)

APN, Aminopeptidase N; CA19-9, carbohydrate antigen 19-9; IQR, interquartile range; SD, standard deviation.

Supplementary Table S3: Association between serum APN/CD13 levels and clinicopathological characteristics in pancreatic cancer

**Characteristics**	**All (*n*=204)**	**Serum APN/CD13 (U/mL)**		***P*-value**
		**High level (≥16.8), *n*=171**	**Low level (<16.8), *n*=33**	
Age (y)				0.351
≥60	126	108	18	
<60	78	63	15	
Gender				0.111
Male	130	113	17	
Female	74	58	16	
Tumor location				0.368
Head	149	127	22	
Body/tail	55	44	11	
Tumor size				0.205
<5 cm	122	99	23	
≥5 cm	82	72	10	
Tumor status				0.287
T1	50	41	9	
T2	62	50	12	
T3	40	32	8	
T4	52	48	4	
Nodal status				0.182
N0	72	57	15	
N1	132	114	18	
Metastatic status				0.120
M0	137	111	26	
M1	67	60	7	
TNM stage				**0.012**
I	23	16	7	
II	65	50	15	
III	49	42	7	
IV	67	63	4	

P-values were determined by Chi-square test.

Supplementary Table S4: Concentrations of serum APN/CD13 in different pancreatic cancer stage groups

Group	No.	Median (IQR)(U/mL)	Mean (SD)(U/mL)
HC	87	9.3 (6.6–12.4)	9.7 (4.8)
Stage I	23	15.2 (9.4–40.8)	24.4 (20.1)
Stage II	65	36.4 (24.9–45.9)	36.0 (15.9)
Stage III	49	54.3 (19.9–62.3)	46.3 (24.9)
Stage IV	67	57.4 (45.2–75.4)	57.0 (26.6)

HC, healthy control; IQR, interquartile range; SD, standard deviation.

Supplementary Table S5: Diagnostic parameters for serum APN/CD13 in the differential diagnosis of pancreatic cancer at different stages *vs.* healthy controls

	AUC (95% CI)	Sensitivity (%)	Specificity (%)	PPV (%)	NPV (%)	Positive LR	Negative LR
HC *vs*. Stage I							
	0.930 (0.861–0.998)	85.7	84.8	83.3	96.5	5.638	0.169
HC *vs*. Stage II							
	0.965 (0.933–0.998)	92.3	94.3	92.3	94.3	16.192	0.082
HC *vs*. Stage III							
	0.937 (0.887–0.986)	89.8	90.8	85.0	95.2	9.761	0.112
HC *vs*. Stage IV							
	0.941 (0.897–0.985)	89.6	93.1	90.8	91.0	12.986	0.112

HC, healthy control; AUC, area under curve; PPV, positive predictive value; NPV, negative predictive value; LR, likelihood ratio; CI, confidence interval.

Supplementary Table S6: Factors associated with one-year mortality of pancreatic cancer patients

Characteristic	All (*n*=196)	Non-survivors (*n*=62)	Survivors (*n*=134)	*P*-value
Age (y)				0.203
≥60	120	42	78	
<60	76	20	56	
Gender				0.154
Male	125	44	81	
Female	71	18	53	
Tumor location				0.122
Head	144	50	94	
Body/tail	52	12	40	
Tumor size				0.351
<5 cm	120	35	85	
≥5 cm	76	27	49	
Tumor status				0.454
T1	47	12	35	
T2	61	17	44	
T3	38	14	24	
T4	50	19	31	
Nodal status				**0.039**
N0	71	16	55	
N1	125	46	79	
Metastatic status				**0.011**
M0	132	34	98	
M1	64	28	36	
TNM stage				**0.007**
I	22	4	18	
II	63	13	50	
III	47	15	32	
IV	64	30	34	
Serum APN/CD13 level				**0.004**
High APN/CD13	165	59	106	
Low APN/CD13	31	3	28	

P-values were determined by Chi-square test.

Supplementary Table S7: Univariate and multivariate analysis of overall survival in patients with pancreatic cancer (Cox proportional hazards regression model)

Characteristics	Univariate analysis		Multivariate analysis	
	HR (95% CI)	*P*-value	HR (95% CI)	*P*-value
Age (≥60 years *vs*. <60 years)	1.24 (0.91–1.73)	**0.043**	1.46 (1.05–3.79)	0.091
Gender (male *vs*. female)	2.81 (1.16–4.96)	0.078		
Tumor location (head *vs*. body/tail)	0.87 (0.53–1.36)	0.283		
Tumor size (≥5 cm *vs*. <5 cm)	1.26 (0.81–1.75)	0.187		
Tumor status (T4/T3 *vs*. T2/T1)	2.31 (1.28–5.83)	0.084		
Nodal status (N1 *vs*. N0)	2.47 (1.31–5.09)	**0.035**	1.73 (0.79–4.02)	0.076
Metastatic status (M1 *vs*. M0)	2.85 (1.66–5.45)	**0.008**	1.36 (0.67–3.52)	**0.025**
TNM stage (IV/III *vs*. II/I)	3.51 (2.06–5.93)	**0.007**	1.63 (0.97–3.59)	**0.021**
CA19-9 level (≥43.3 U/mL *vs*. <43.3 U/mL)	3.32 (1.22–5.64)	**0.010**	1.44 (0.71–3.73)	**0.041**
Serum APN/CD13 level (≥16.8 U/mL *vs*. <16.8 U/mL)	4.17 (2.05–8.20)	**0.001**	3.12 (1.82–5.97)	**0.013**
